# Recurrent wheezing in neonatal pneumonia is associated with combined infection with Respiratory Syncytial Virus and *Staphylococcus aureus* or *Klebsiella pneumoniae*

**DOI:** 10.1038/s41598-018-19386-y

**Published:** 2018-01-17

**Authors:** Qin Zhong, Hui Feng, Qi Lu, Xu Liu, Qi Zhao, Yue Du, Xian-Hong Zhang, Jia-Rong Wang

**Affiliations:** 1 Department of Neonatology, Children’s Hospital of Chongqing Medical University, Ministry of Education Key Laboratory of Child Development and Disorders, Chongqing, 400014 China; 2 China International Science and Technology Cooperation base of Child development and Critical Disorders, Chongqing, 400014 China; 3 Chongqing Key Laboratory of Pediatrics, Chongqing, 400014 China; 4 Qianjiang Central Hospital of Chongqing, chongqing, 409000 China

## Abstract

Both viral and bacterial infections can be associated with wheezing episodes in children; however, information regarding combined infections with both viral and bacterial pathogens in full term neonates is limited. We sought to investigate the effects of viral–bacterial codetection on pneumonia severity and recurrent wheezing. A retrospective cohort study was conducted on neonates admitted to our hospital with pneumonia from 2009 to 2015. Of 606 total cases, 341 were diagnosed with RSV only, and 265 were diagnosed with both RSV and a potential bacterial pathogen. The leading four species of bacteria codetected with RSV were *Escherichia coli*, *Klebsiella pneumoniae*, *Staphylococcus aureus* and *Enterobacter cloacae*. Neonates with RSV and a potential bacterial pathogen were significantly more likely to have worse symptoms, higher C-reactive protein values and more abnormal chest x-ray manifestations with Bonferroni correction for multiple comparisons (*P* < 0.01). On Cox regression analysis, an increased risk of recurrent wheezing was found for neonates positive for RSV–*Staphylococcus aureus* and RSV–*Klebsiella pneumoniae*. Our findings indicate that the combination of bacteria and RSV in the neonatal airway is associated with more serious clinical characteristics. The presence of RSV and *Staphylococcus aureus* or *Klebsiella pneumoniae* may provide predictive markers for wheeze.

## Introduction

Pneumonia is the primary infectious cause of death worldwide in children under five years of age. The greatest risk of death from pneumonia occurs in the neonatal period^1,2^. It is estimated that 0.136 million neonatal deaths were caused by pneumonia in 2013^3^.

Some studies have reported increasing detection rates for mixed viral–bacterial infections in children with community-acquired pneumonia^4,5^ and have also found an interplay between viruses and bacteria. Viral respiratory infections can elevate nasopharyngeal bacterial colonization density and promote bacterial infections in children^6–8^. In addition, respiratory viruses play an important role in wheezing episodes which may mark the beginning of asthma^9–11^. Recently, bacterial colonization has also been found to be associated with wheezing and asthma^6,12^. Other research has focused primarily on children with low birth weights^13^, whereas the interaction of pulmonary viruses and bacteria are relatively unclear in full term neonates. Nevertheless, it has been hypothesized that adverse exposures in early postnatal life might influence lung growth and development, and lead to persistently smaller airways and impaired lung function. Therefore, in this study, we investigated the effect of viral–bacterial codetection on pneumonia severity in full term neonates, and sought to determine if codetection increases the risk of recurrent wheezing, rhinitis and eczema.

## Patients and Methods

### Patients

This was a retrospective cohort study and conducted at Children’s Hospital of Chongqing Medical University, a tertiary university hospital that has a 214-bed neonatal unit with an annual admission rate of over 6000 neonates. Inclusion criteria included the following: 1) hospitalized neonates (≤28 days of age) with an admitting diagnosis of clinical pneumonia between Jan 2009 and Dec 2015, and 2) respiratory samples collected within 24 hours of admission were positive for a viral respiratory pathogen. Exclusion criteria included the following: (1) premature infants (gestational age <37 weeks at birth); and (2) neonates with congenital malformation, immunodeficiency, severe haemolytic disease, or severe malnutrition. Clinical pneumonia was defined by the presence of a clinical sign (such as cough, wheeze, phlegm production, tachypnoea, cyanosis, or fever) and rales or rhonchi on chest auscultation^14,15^. Chest x-ray showing lung consolidation or infiltrate was used to diagnose pneumonia. We followed up the included neonates until December 31, 2016. Follow-up times ranged from one to seven years. Patients with incomplete data were excluded from data analysis.

This study was approved by the Ethics Committee of Children’s Hospital of Chongqing Medical University (file number: 62/2017). Informed consent was obtained from the parents when patients were admitted to the hospital. All methods were performed in accordance with the relevant guidelines and regulations.

## Methods

### Sample collection

Nasopharyngeal aspirates were taken for viral testing^16^, and sputum samples were obtained for bacterial culture^6^. Within 2 hours of collection, the samples were transported to the microbiology laboratory, and the sputum samples were cultured on chocolate and blood agar plates for bacteria. Viral antigens for respiratory syncytial virus (RSV); adenovirus (ADV); influenza virus A (IVA); influenza virus B (IVB); and parainfluenza virus (PIV) I, PIV II, and PIV III were tested for using direct immunofluorescence^16^. Codetection was defined as a positive detection of >1 clinically–relevant microbe.

### Data collection

Data on patient demographics, microbiology results, laboratory parameters and chest x-ray findings were obtained from the hospital electronic medical records system. We used questionnaires to obtain information on the subsequent development of recurrent wheezing, rhinitis and eczema during the first 3 years of life. The questionnaires were based on the Japanese version of the International Study of Asthma and Allergies in Childhood (ISAAC) Phase Three Questionnaire^17^. During the follow-up period, we called parents and asked them questions according to the questionnaire. Wheeze was defined as wheezing or whistling sounds, persistent troublesome cough or breathlessness^6^. A wheezing episode was defined as a respiratory episode with wheezing for more than 1 day. The interval between two episodes was defined as a period of at least 7 days without respiratory symptoms. Recurrent wheezing was defined as having three or more episodes of wheezing^18^. Survival time of wheeze for each child was defined as time from neonatal pneumonia to the first occurrence of wheeze.

### Statistical analysis

Continuous variables were analysed using the Mann–Whitney U test because data were not normally distributed. Categorical variables were analysed using the chi-square test, and multiple comparisons were adjusted using the Bonferroni correction (*P* < 0.01). The association between codetection of virus with bacteria, covariates, and subsequent development of recurrent wheezing, rhinitis and eczema were assessed by logistic regression analysis to estimate odds ratios (ORs). Considering the inconsistent follow-up time, survival statistics were used to analyse the risk of recurrent wheezing. The cumulative risks of recurrent wheezing during the first 3 years stratified according to codetection by RSV and bacteria were estimated by the Kaplan–Meier estimator. Changes in risk were quantified by Cox regression.

Continuous variables were reported as medians (25th–75th percentiles), and categorical variables as frequencies (percentages). Statistical analyses were performed using the IBM Statistical Package for Social Sciences (SPSS) version 21. *P* < 0.05 was considered statistically significant.

### Data availability statement

Requests for materials should be addressed to Q.L (email: qilu_qi@163.com)

## Results

During the study period, a total of 27,169 neonates were admitted to the hospital for clinical pneumonia. Of these, 8128 (29.9%) had nasopharyngeal aspirates collected to check for respiratory viruses within 24 hours of admission.

### Detections and Codetections

**Figure 1:**
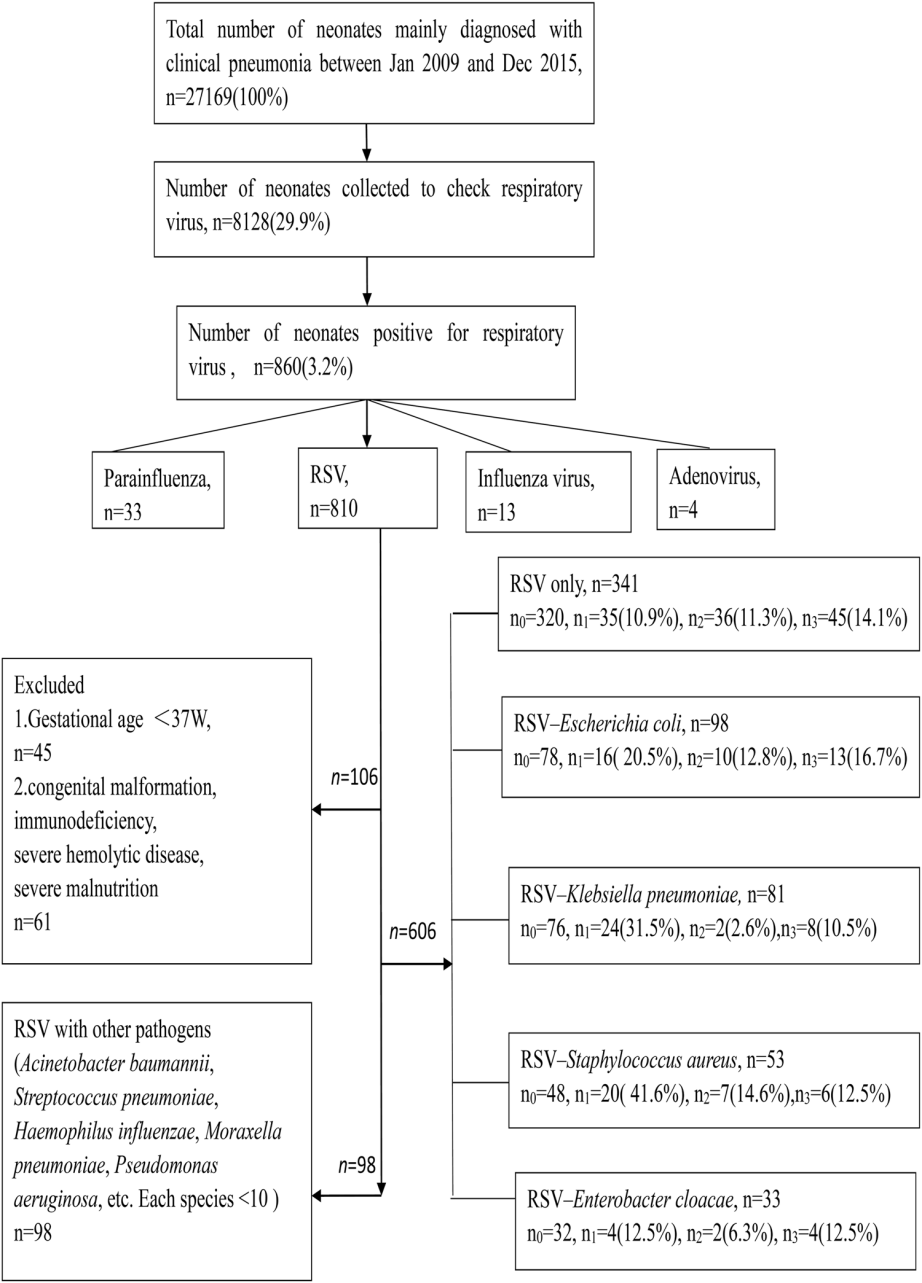
Enrollment and outcomes. We studied 606 neonates, 52 (8.6%) neonates were lost to follow up, 554 neonates had follow-up results. n0 = number of neonates who were followed up, n1 = number of neonates who had recurrent wheezing. n2 = number of neonates who had allergic rhinitis, n3 = number of neonates who had eczema.

Among the 860 cases with positive virus detection, RSV (810; 94.2%) was most frequently detected. In RSV-positive patients, *Escherichia coli* (98/12.1%), *Klebsiella pneumoniae* (81/10.0%), *Staphylococcus aureus* (53/6.5%) and *Enterobacter cloacae* (33/4.1%) were the most commonly detected bacteria (Fig. 1).

Other viruses were identified in 50 cases, including 33 samples with parainfluenza virus, 13 with influenza virus and 4 with adenovirus. *Escherichia coli* was codetected with these viruses more often than other bacteria, with a total of 11 cases.

606 neonates were followed up, and 554 (91.4%) of them had follow-up results during the first 3 years of life.

### Clinical Characteristics

In consideration of sample size, RSV-positive cases were further analysed. We assessed whether the main four species of codetected bacteria in RSV-positive neonates influenced disease severity. There were no significant differences in the baseline characteristics of age, sex, weight, gestational age at birth, birth weight, and caesarean section rate between RSV-positive neonates with and without bacterial pathogen codetection with *Escherichia coli*, *Klebsiella pneumoniae*, *Staphylococcus aureus*, or *Enterobacter cloacae*. Furthermore, the rates of nasal obstruction, cough, cyanosis, moist rales and diarrhoea did not differ between the groups.

Neonates with codetection of RSV and a potential bacterial pathogen were significantly more likely to present with shortness of breath (*P* < 0.001), wheezing (*P* = 0.008), chest retraction (*P* = 0.009) and a higher oxygen requirement (*P* < 0.001), higher C-reactive protein values (*P* < 0.001) and more abnormal chest x-rays (*P* < 0.001) than those with detection of RSV only.

Neonates with codetection of RSV and *Staphylococcus aureus* had higher white blood cell counts, higher blood platelet counts, and a higher incidence of fever. However, clinical symptoms and laboratory tests were not significantly different between the RSV only and the RSV–*Enterobacter cloacae* group.

After adjustment for multiple comparisons by Bonferroni correction (P < 0.01), neonates with RSV and a potential bacterial pathogen were significantly more likely to have worse symptoms, higher C-reactive protein values and more abnormal chest x-ray manifestations (Table 1).

**Table 1:** Demographic, clinical symptoms and laboratory parameters in neonates with RSV only and RSV codetected with bacteria.

	RSV only n = 341	RSV codetected with bacteria n*=265*	*P*_0_ value	RSV–*Escherichia coli* n = 98	*P*_1_ value	RSV–*Klebsiella pneumonia* n = 81	*P*_2_ value	RSV–Staphylococcus aureus n = 53	*P*_*3 ‘*_value	RSV–*Enterobacter cloacae* n = 33	*P*_4_ value
Demographic Data											
Age (day)	15 [11–20]	16 [12–21]	0.956	15 [12–19]	0.987	16 [12.5–20.5]	0.895	13 [11–18]	0.575	17 [12.5–20]	0.549
Gestational age at birth(week)	39 [38.2–39.6]	39 [38–40]	0.348	39 [38.2–39.5]	0.582	38 [37.4–38.7]	0.473	39 [38.3–39.7]	0.671	39 [38.5–39.6]	0.739
Sex (male,%)	188 (55.1)	154 (58.1)	0.463	58 (59.2)	0.476	53 (65.4)	0.092	27 (50.9)	0.569	16 (48.5)	0.464
Weight(g)	3600 [3100–4064]	3580 [3160–3920]	0.491	3503 [3200–3843]	0.374	3409 [3200–3605]	0.328	3640 [3335–3943]	0.834	3451 [3150–3798]	0.503
Birth weight(g)	3320 [3020–3590]	3300 [3000–3600]	0.996	3263 [3050–3718]	0.885	3300 [3075–3550]	0.997	3350 [3100–3680]	0.968	3250 [3000–3625]	0.516
Caesarean section (n,%)	215 (63.0)	160 (60.4)	0.502	52 (53.1)	0.074	53 (65.4)	0.689	35 (66.0)	0.674	20 (60.6)	0.781
Oxygen requirement (n,%)	144 (42.2)	158 (59.6)	**<0.001**	58 (59.2)	**0.003**	47 (58.0)	0.01	36 (67.9)	**<0.001**	17 (51.5)	0.304
Clinical Symptoms (n,%)											
Nasal obstruction	172 (50.4)	152 (57.4)	0.09	53 (54.1)	0.525	49 (60.5)	0.103	31 (58.5)	0.275	19 (57.6)	0.434
Cough	266 (78.0)	222 (83.8)	0.075	80 (81.6)	0.439	69 (85.2)	0.151	45 (84.9)	0.252	28 (84.8)	0.46
Shortness of breath	233 (68.3)	220 (83.0)	**<0.001**	78 (79.6)	0.031	67 (82.7)	0.01	49 (92.5)	**<0.001**	26 (78.8)	0.214
Cyanosis	237 (69.5)	200 (75.5)	0.104	75 (76.5)	0.176	64 (79.0)	0.089	36 (67.9)	0.817	25 (75.8)	0.454
Moist rale	126 (37.0)	116 (43.8)	0.089	42 (42.9)	0.289	37 (45.7)	0.147	21 (39.6)	0.708	16 (48.5)	0.192
Wheezing	53 (15.5)	64 (24.2)	**0.008**	22 (22.4)	0.109	21 (25.9)	0.027	15 (28.3)	0.022	6 (18.2)	0.691
Chest retraction	83 (24.3)	90 (34.0)	**0.009**	33 (33.7)	0.065	29 (35.8)	0.036	19 (35.8)	0.075	9 (27.3)	0.709
Fever	63 (18.5)	69 (26.0)	0.025	25 (25.5)	0.125	16 (19.8)	0.791	21 (39.6)	**<0.001**	7 (21.2)	0.7
Diarrhoea	90 (26.4)	81 (30.6)	0.258	37 (37.8)	0.029	22 (27.2)	0.888	14 (26.4)	0.997	8 (24.2)	0.789
Laboratory tests											
WBC/mm^3^	8.2 [7.2–9.4]	10.0 [9.6–11.1]	0.172	9.8 [8.7–11.0]	0.163	10.2 [8.9–11.2]	0.089	11.7 [10.4–13.1]	**0.008**	9.6 [8.5–10.9]	0.243
Eosinophils %	3 [2–4]	2 [3–5]	0.514	3.5 [2–4.5]	0.681	3.2 [2.2–4.3]	0.895	3.7 [2.5–4.7]	0.456	2.8 [2–3.8]	0.873
Platelets/mm^3^	305 [255–358]	379 [303–451]	0.023	368 [310–420]	0.103	393 [330–455]	0.02	420 [366–485]	**0.004**	372 [310–430]	0.087
Higher CRP (n,%)	32 (9.4)	57 (21.5)	**<0.001**	20 (20.4)	**0.003**	16 (19.8)	**0.008**	15 (28.3)	**<0.001**	6 (18.2)	0.11
Abnormal chest x-ray (n,%)	233 (68.3)	224 (84.5)	**<0.001**	80 (81.6)	0.01	71 (87.7)	**<0.001**	47 (88.7)	**0.002**	26 (78.8)	0.214

WBC: white blood cell counts. Higher CRP:C-reactive protein levels were measured by scatter rate turbidity comparison, ≥8 mg/L.

Abnormal x-ray: emphysema, lobar consolidation, atelectasis.

Data expressed in median values with 25–75% interquartile ranges. Numbers in parenthesis represent the percentage of subjects in each group.

*P*_0_ value: comparisons between RSV only and RSV with bacteria; *P*_1_ value: comparisons between RSV only and RSV–*Escherichia coli*;

*P*_2_ value: comparisons between RSV only and RSV–*Klebsiella pneumoniae*; *P*_3_ value: comparisons between RSV only and RSV–*Staphylococcus aureus*;

*P*_4_ value: comparisons between RSV only and RSV–*Enterobacter cloacae*. Significant *P* values are in bold.

Mann-Whitney U test was used for continues variables and Chi-square test for analyses of categorical variables; the Bonferroni correction was applied for multiple comparisons (*P* < 0.01).

### Codetection in relation to recurrent wheezing, rhinitis and eczema

**Figure 2:**
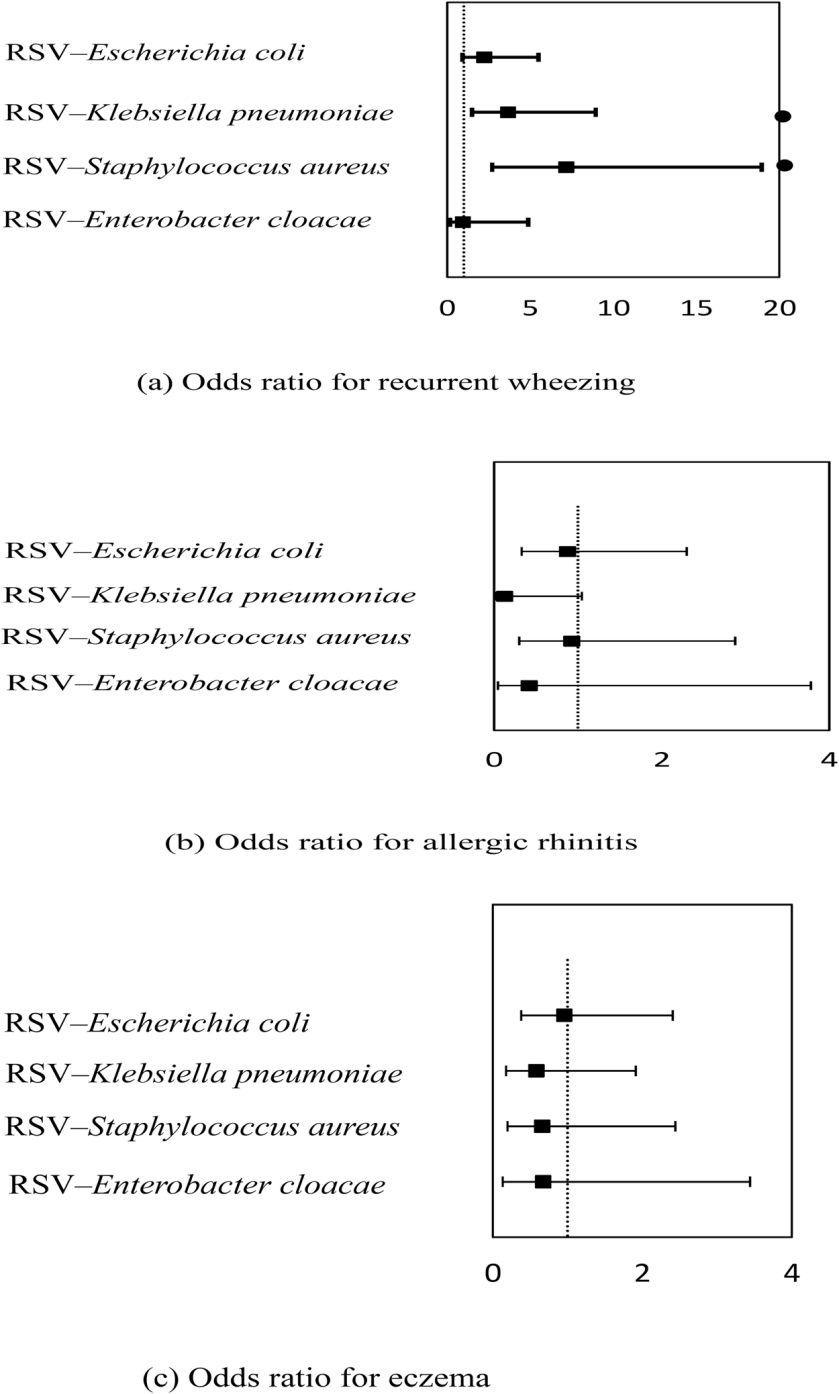
Odds ratio for recurrent wheezing, allergic rhinitis and eczema. Logistic regression analysis: According to the results of telephone interview of RSV only and RSV with bacteria. Odds ratios were adjusted for the following possible confounders: sex, caesarean section, allergic history, family history of allergies, educational background of mother, living conditions, antibiotic therapy before admission, feeding option, follow-up time, age, gestational age at birth, weight, birth weight. (^●^P<0.05).

After controlling for confounding variables with logistic regression analysis, neonates with codetection of RSV–*Staphylococcus aureus* and RSV–*Klebsiella pneumoniae* had increased risks of recurrent wheezing (OR 7.16, 95% CI, 2.70–18.94, *P* < 0.001 and OR 3.66; 95% CI, 1.49–9.94, *P* = 0.005; respectively) compared with those with RSV only. There was no statistically significant difference among the groups in the risks of rhinitis and eczema (Fig. 2).

### Survival analyses for risk factors associated with recurrent wheezing during the first 3 years of life

The risk of recurrent wheezing was increased in neonates with codetection of RSV–*Staphylococcus aureus* and RSV–*Klebsiella pneumoniae*, but not in those with codetection of RSV–*Escherichia coli* or RSV–*Enterobacter cloacae* (Table 2). Those with codetection of RSV–*Staphylococcus aureus* were 4.22 times more likely to have recurrent wheezing than RSV only cases (HR, 4.22; 95% CI, 2.02–8.83). The hazard ratio for the codetection of RSV–*Klebsiella pneumoniae* was 3.15 (95% CI, 1.51–6.57) for recurrent wheezing.

**Table 2:** Risk of recurrent wheezing in Cox regression analysis.

Risk factors	Recurrent wheezing = 99	*P* value	HR	95% CI
	N_w_ (%)			
Male (N_t_ = 320)	73 (73.7)	**0.014**	2.14	1.17–3.91
Caesarean section (N_t_ = 347)	63 (63.6)	0.688	1.12	0.64–1.95
Allergic history (N_t_ = 39)	13 (13.1)	**0.008**	2.97	1.33–6.63
Family history of allergies (N_t_ = 55)	19 (19.2)	**0.037**	2.1	1.05–4.22
Mother with bachelor degree or above (N_t_ = 164)	36 (36.4)	0.123	1.75	0.86–3.56
Living in the city (N_t_ = 370)	63 (63.6)	0.152	0.61	0.31–1.20
Antibiotic therapy before admission (N_t_ = 259)	43 (43.4)	0.94	0.98	0.56–1.72
*Type of feedings*				
Breastfed (N_t_ = 213)	NA	NA	NA	NA
Formula fed (N_t_ = 130)	19 (19.2)	0.178	0.6	0.29–1.26
Mixed feedings (N_t_ = 211)	31 (31.3)	0.064	0.55	0.29–1.04
*Detected pathogens*				
RSV only (N_t_ = 320)	NA	NA	NA	NA
RSV–*Escherichia coli* (N_t_ = 78)	16 (16.2)	0.089	1.95	0.90–4.19
RSV–*Klebsiella pneumoniae* (N_t_ = 76)	24 (24.2)	**0.002**	3.15	1.51–6.57
RSV–*Staphylococcus aureus* (N_t_ = 48)	20 (20.2)	**<0.001**	4.22	2.02–8.83
RSV–*Enterobacter cloacae* (N_t_ = 32)	4 (4.0)	0.967	1.03	0.24–4.51
<7 days of age (N_t_ = 37)	10 (10.1)	0.217	1.79	0.71–4.51
Only child of the family (N_t_ = 377)	70 (70.7)	0.616	1.21	0.58–2.51
Maternal age <30 years (N_t_ = 408)	68 (68.7)	0.127	0.6	0.31–1.16

N_w_ = the number of neonates who had the risk among recurrent wheezing neonates.

N_t_ = the number of neonates who had the risk among total neonates who had follow-up results; NA: not applicable.

HR: hazard ratio; 95% CI: 95% confidence interval. Significant values are in bold.

Cox regression model: the outcomes were adjusted for all the listed risk factors.

**Figure 3:**
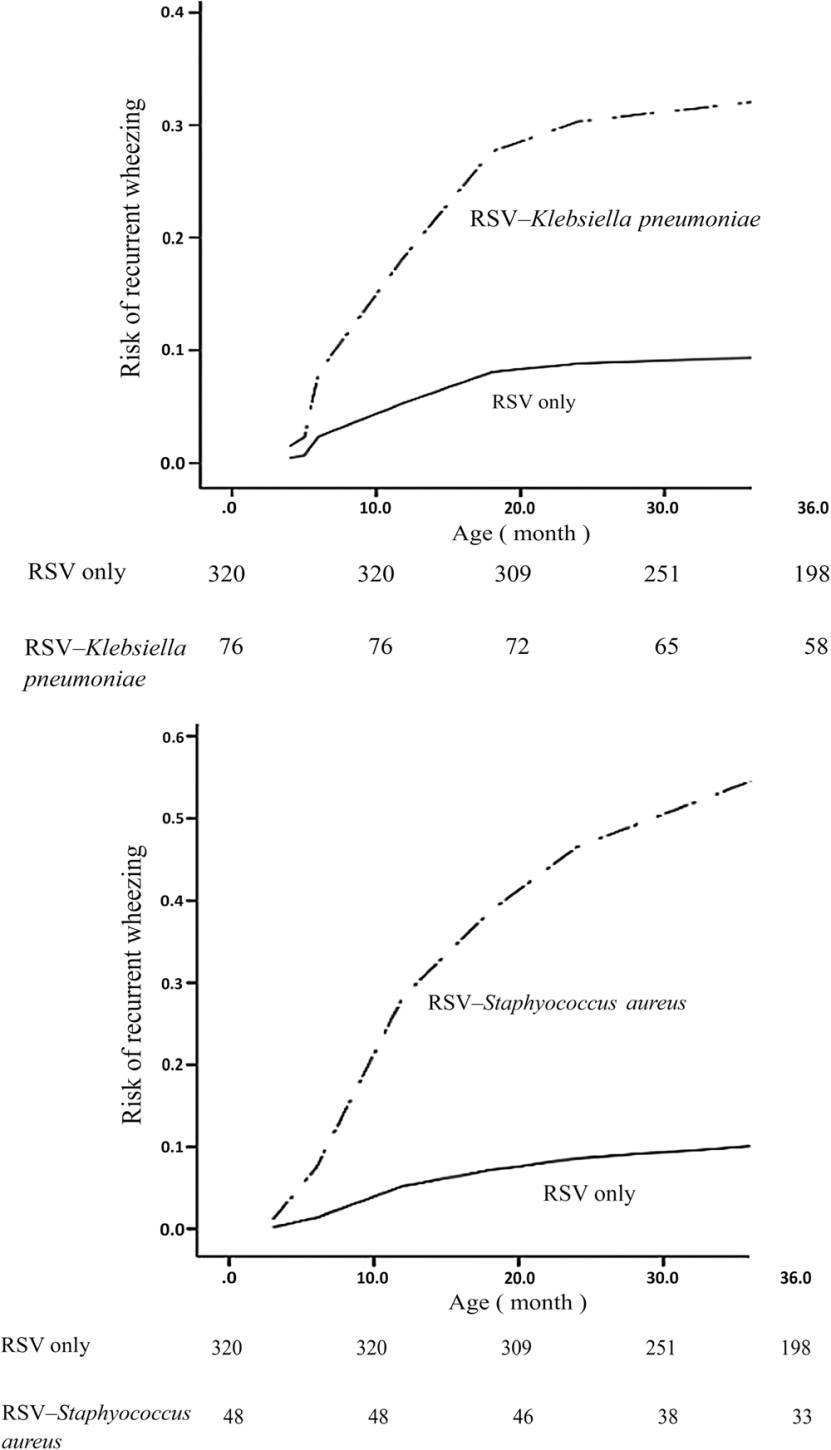
Cumulative risk of recurrent wheezing for neonates with RSV only VS RSV–Klebsiella pneumoniae and RSV only VS RSV–*Staphylococcus aureus*.

In the Kaplan–Meier analysis, the risk of recurrent wheezing associated with codetection of RSV–*Staphylococcus* and RSV–*Klebsiella pneumoniae* continually increased during the first 3 years of life (Fig. 3). There was no significant difference between the risks of recurrent wheezing for RSV–*Klebsiella pneumoniae* and RSV–*Staphylococcus aureus*.

## Discussion

RSV was the predominant viral pathogen seen in our study, consistent with results from other studies^19,20^. RSV was detected in 10% of neonatal pneumonia samples, however, other studies have shown RSV to be present in about 22%–29% of all acute lower respiratory tract infection cases^19,20^. This could be dueto the fact that we only included full term infants in our study. Infants are most susceptible to RSV infections at 6–11 months of age, when they lack protection from maternal immunoglobulins^19^.

Several previous studies have shown an association between viruses and bacteria. Hishiki *et al*. reported that *Haemophilus influenzae*, *Streptococcus pneumoniae* and *Moraxella catarrhalis* were most frequently isolated from paediatric inpatients having RSV bronchopulmonary infection in Japan^21^. Menno R *et al*. observed a positive association between RSV and *Haemophilus influenzae* in the upper respiratory tract of children <2 years of age in the Netherlands^22^. Our findings differed from those studies, showing RSV was more likely to be codetected with *Escherichia coli*, *Klebsiella pneumoniae*, *Staphylococcus aureus* and *Enterobacter cloacae*. This may be due to differences in geography and population, as the most common gram-negative organisms in China are *Escherichia coli*, *Klebsiella pneumoniae* and *Haemophilus influenzae*, and the most common gram-positive organism is *Staphylococcus aureus*^14^.

In our study, codetection of RSV and potential bacterial pathogens contributed to enhanced symptoms of disease, higher C-reactive protein values and more abnormal chest x-ray manifestations. These findings suggest that during RSV infection, these specific bacterial pathogens were cofactors that contributed to the severity of respiratory symptoms and inflammatory reactions. One possible explanation for this pattern was that virally induced inflammation might alter bacterial gene expression, leading to a more pathogenic phenotype, increased bacterial virulence and decreased clearance of bacteria^23,24^.

The incidence of wheezing is quite high in the first 3 years of life^25^, and RSV might be the initiator (post-RSV wheezing disorder). Notably, there is increasing evidence that the combination of specific bacteria and rhinovirus is associated with wheezing and asthma. Previous studies showed that the detection of rhinovirus together with *M. catarrhalis* and *S. pneumoniae* increased the risk of asthma exacerbations in children 4–12 years of age^26^. In our study, cases with codetection of *Staphylococcus aureus* and *Klebsiella pneumoniae* with RSV were associated with an increased risk of recurrent wheezing compared with RSV only cases in children from birth to age 3. This may result from the fact that specific bacteria are involved in allergic inflammation and may induce immunomodulatory effects and a Th2-type of eosinophilic inflammation in patients with allergic disease^12,27^.

To our knowledge, this study is the first to research both viral and bacterial detection in full term neonates, and we found a relationship between RSV and specific bacteria. However, some potential study limitations need to be discussed. First, single-centre retrospective cohort analysis has known inherent limitations, and further prospective multi-centre studies are recommended. Second, we had no information on viral and bacterial loads, and quantitative analysis could not be performed in our study. Third, sicker neonates were more likely to have hospital-associated acquisition of bacteria, and, therefore, the bacteria could simply be a marker of disease severity and sequelae. Furthermore, it is worth noting that detection of bacteria in sputum samples might not indicate causative agents. Bacteria may be contaminants from the nasopharynx, because healthy infants often carry pathogenic bacteria there. Thus, because we must be cautious in using the terms ‘causative agent’ or definite ‘coinfection’, we used ‘codetected’ to state the relationship between the detected bacteria and viruses in our study.

## Conclusion and Prospection

The combination of bacteria and RSV in the neonatal airway was found to be associated with an additive effect that resulted in more serious clinical symptoms. The presence of RSV and *Staphylococcus aureus* or *Klebsiella pneumoniae* in the airway were associated with an increased risk of recurrent wheezing in early life and may provide a predictive marker for wheezing.
